# Determining Spatial Variability of Elastic Properties for Biological Samples Using AFM

**DOI:** 10.3390/mi14010182

**Published:** 2023-01-11

**Authors:** Stylianos Vasileios Kontomaris, Andreas Stylianou, Georgios Chliveros, Anna Malamou

**Affiliations:** 1 BioNanoTec Ltd., Nicosia 2043, Cyprus; 2 Faculty of Engineering and Architecture, Metropolitan College, 15125 Athens, Greece; 3 School of Sciences, European University Cyprus, Nicosia 2404, Cyprus; 4 School of Electrical and Computer Engineering, National Technical University of Athens, 15780 Athens, Greece

**Keywords:** Young’s modulus maps, nanoscale, biomaterials, cells, disease diagnosis, advanced microscopy

## Abstract

Measuring the mechanical properties (i.e., elasticity in terms of Young’s modulus) of biological samples using Atomic Force Microscopy (AFM) indentation at the nanoscale has opened new horizons in studying and detecting various pathological conditions at early stages, including cancer and osteoarthritis. It is expected that AFM techniques will play a key role in the future in disease diagnosis and modeling using rigorous mathematical criteria (i.e., automated user-independent diagnosis). In this review, AFM techniques and mathematical models for determining the spatial variability of elastic properties of biological materials at the nanoscale are presented and discussed. Significant issues concerning the rationality of the elastic half-space assumption, the possibility of monitoring the depth-dependent mechanical properties, and the construction of 3D Young’s modulus maps are also presented.

## 1. Introduction

Atomic Force Microscopy (AFM) was developed in 1986 by Binnig and colleagues and is part of the scanning probe microscopy (SPM) family [1]. AFM records the interaction between a sharp tip at the end of a cantilever and a sample of interest [2]. One of the fundamental advantages of AFM compared with other microscopy techniques is the simultaneous imaging [2] and mechanical characterization at the nanoscale [3]. Additionally, AFM can be used to test biological samples very close to physiological conditions; for example, special treatment of the sample is not necessary (as in scanning electron microscopy), and foremost it can operate with samples in liquid conditions [3].

Regarding mechanical nanocharacterization, the basic operation principle is to apply a well-defined force on the specimen and monitor the piezo-displacement of the sample’s surface when moving toward the tip. The indentation is the difference between the piezo-displacement of the sample of interest and the piezo-displacement of a hard sample in which no indentation occurs (using the same applied force) [4]. The force-indentation data are fitted to appropriate models from contact mechanics depending on the shape of the indenter and the sample [5]. Thus, Young’s modulus can be calculated, also referred to as the ‘apparent Young’s modulus’. If this procedure is repeated on many different nanoregions, mechanical nanocharacterization over extended areas of a sample can be acquired. Therefore an “elasticity spatial distribution” or a “Young’s modulus map” can be constructed [6]. It is significant to note that the accuracy of the AFM indentation method strongly depends on the calibration of the AFM tip [7,8]. The nominal values of the tip radius and the cantilever’s spring constant provided by the manufacturers depict a large variation deviation ~100% for individual, non-calibrated probes [8].

The mechanical characterization of biological samples at the nanoscale is crucial since it unravels fundamental information about pathological conditions such as cancer or osteoarthritis [3]. In particular, cancer progression is related to changes in the mechano-cellular phenotype that are reflected in changes in the mechanical properties of the tumor microenvironment [6]. AFM revealed that, in most cases, cancer cells are significantly softer compared with healthy ones due to cytoskeletal alterations [6,9]. On the other hand, cancer tissues are stiffer compared with normal ones due to the overexpression of extracellular matrix (ECM) proteins, mainly collagen type I [10]. Thus, AFM revealed that in the case of normal/benign tissues, the stiffness distribution consists of one single peak, while in cancer tissues, at least two different peaks are observed due to the softening of the cancerous cells (Lower Elasticity Peak-LEP) and the stiffening of the surrounding tissue (Higher Elasticity Peak-HEP) [6]. 

AFM’s contribution is also very important for the early diagnosis of osteoarthritis [11,12]. In particular, articular cartilage has different behavior depending on the scale that is being tested [11]. At the microscale, it behaves as a uniform, non-structured material [11] and can be approximated to be a poroviscoelastic material. Thus, the overall stiffness measurements provide an aggregate modulus, both viscous and elastic [12]. Stolz et al. (2009) proved that at the early stages of osteoarthritis, the micro-stiffness could not provide sufficient information for diagnosis [11]. On the other hand, at the nanoscale, stiffness can be used as a physical parameter to distinguish healthy cartilage from cartilage with osteoarthritis [11]. 

In addition, AFM has also been used to research other pathological conditions, such as Alzheimer’s Disease and HIV [13,14,15,16]. It is important to note that, when it comes to diagnostics, accurate mechanical nanocharacterization is crucial in order to use AFM as a real clinical tool. AFM could become a strong clinical tool in the future if novel developments enable user-independent, accurate diagnosis based on accurate and strict mathematical criteria. However, several drawbacks [5,17] must be overcome in order to use AFM in real clinical practice, as the characterization of biological materials is still a challenging procedure [17,18,19]. 

The aim of AFM research nowadays is a detailed spatial and user-independent nanocharacterization of soft biological samples at the nanoscale. In this review, the basic procedures for a detailed spatial characterization that have been proposed up to date are presented and discussed. The classic Young’s modulus maps and their limitations, the depth-dependent characterization, and recently developed groundbreaking techniques for 3D and 4D characterizations are presented. In addition, the purpose of this review is not only to present the techniques that have been developed for mechanical nanocharacterization but also to provide information regarding the reliability of these methods. 

A question that arises is that as classic contact mechanics models have been derived for linear elastic samples, is it rational to use them for creating Young’s modulus maps at the nanoscale? Biological samples are highly heterogeneous at the *x*-*y* plane (surface) and also depth dependent, i.e., the *z*-axis. Therefore, what is the validity of spatial characterizations at the nanoscale when using classical contact mechanics? In this review, the latest advances regarding a complete mechanical characterization at the nanoscale in three-dimensional space are presented. In Section 2, the elastic half-space assumption when creating a Young’s modulus map is discussed, and the rationality of this approach regarding experiments on biological samples is explained. Furthermore, models to retrieve the elastic and viscoelastic properties of biological samples and corrections for thin samples are presented. In Section 3, the methodologies, and mathematical models for estimating the depth–dependent mechanical properties of biological samples are explained. Section 4 focuses on determining the spatial mechanical properties of cells, and Section 5 on determining the spatial mechanical properties of collagen fibrils. In Section 6, models for 3D and 4D nanocharacterization are presented, and in Section 7, the challenges to overcome are explained. Creating methods of high reliability will be a significant step forward toward the application of AFM and propel it as a novel diagnostic tool for assessing disease diagnosis and for monitoring treatment outcomes.

## 2. Elastic Half Space Assumption and Standard Young’s Modulus Maps

**Figure 1:**
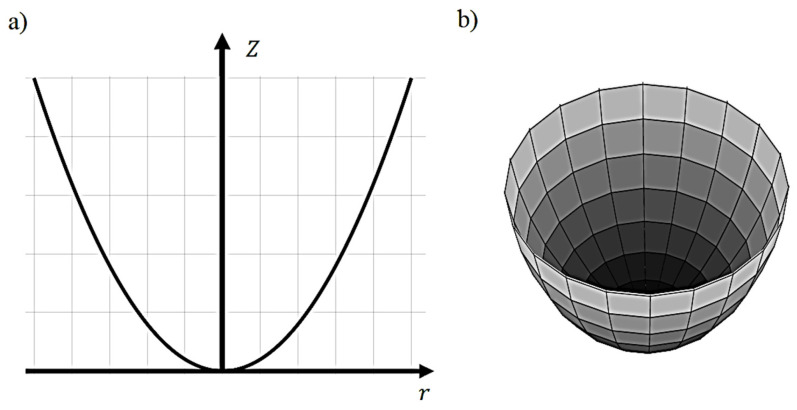
(**a**) A function of the form $Z=Br^{n}$ that is rotated about the *z*-axis to produce a solid of revolution. (**b**) A typical indenter shape that is described using the aforementioned function.

In AFM indentation experiments, biological samples are usually approximated to elastic half-spaces [20]. An elastic half-space is an elastic material that extends infinitely in all directions, including depth, with the surface at the top as the boundary and always behaves linearly [21]. In addition, the pyramidal indenters that are used are approximated to axisymmetric indenters that are described by an arbitrary function $Z=Br^{n}$ (where B, n are constants that depend on the indenter’s shape), which is rotated about the *z*-axis (Figure 1a) to produce a solid of revolution (Figure 1b). The function is chosen so as f(0)=0 with the only restriction to be infinitely differentiable [5]. 

The mechanical properties of a material approximated to an elastic half-space can be determined by fitting the equations from Hertzian mechanics to force indentation data. In that case, the AFM tip can be approximated to an axisymmetric indenter as described above, the force applied on the material (F) with respect to the indentation depth (h) is provided by a general equation of the following form [5,22]:

(1) $$F=ah^{m}$$

In Equation (1), a is a constant parameter that depends on the properties of the material and the shape of the indenter. In particular, in the case of a conical indenter, m=2 and

(2) $$a=\frac{2}{\pi }\frac{Ε}{1-v^{2}}\tan\left(\theta \right)$$

**Figure 2:**
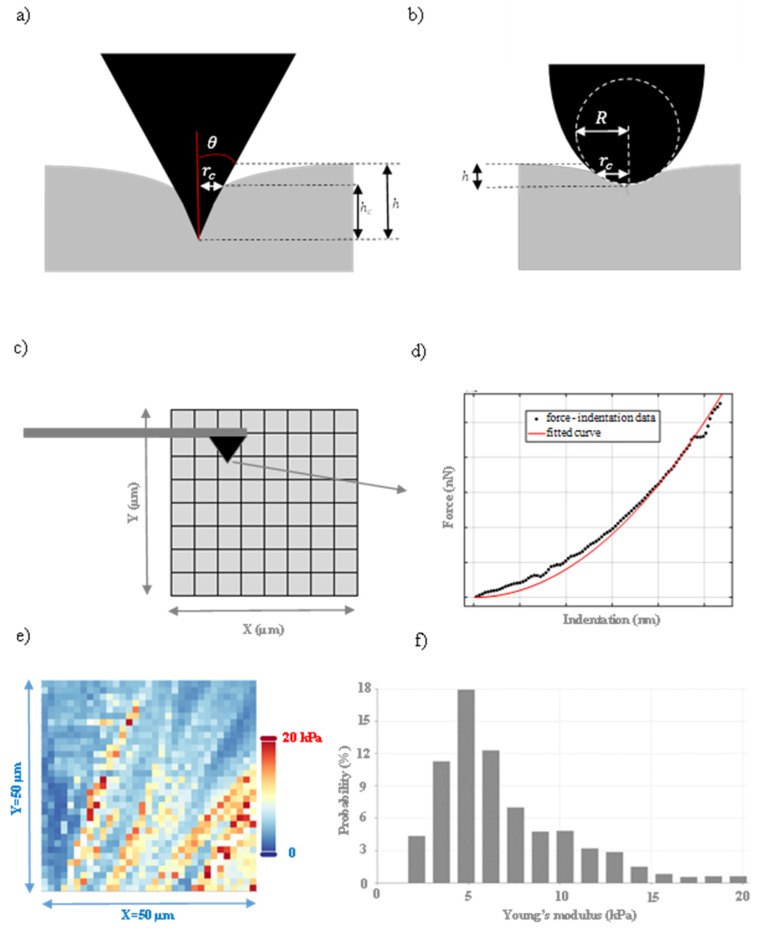
(**a**) A conical and, (**b**) a parabolic indenter, indenting an elastic half space. (**c**) Illustration of the procedure for obtaining a Young’s modulus map. The area of interest is divided to many nanoregions. (**d**) Force–indentation data is obtained on each nanoregion and is fitted to the appropriate model of Hertzian mechanics (Equations (1)–(5)). (**e**) A Young’s modulus map obtained on a fibroblast (using open access nanoindentation data obtained with a conical indenter (cone’s half angle 25° and cantilever’s spring constant 0.01 Nm^−1^) [29]). Only the first 800 nm of the force-indentation data was fitted to Equation (9) to avoid the possibility of a substrate effect. (**f**) The ‘probability-Young’s modulus’ graph. This graph shows the extended heterogeneity of the fibroblast.

In Equation (2), E,v are Young’s modulus and Poisson’s ratio of the sample, respectively, and θ is the cone’s half-angle [22]. A conical indenter, indenting an elastic half-space, is presented in Figure 2a. The radius between the indenter and the sample at contact depth $h_{c}$, is the contact radius $r_{c}$ [23]. For parabolic indenters (Figure 2b) with tip radius R, m=3/2 and

(3) $$a=\frac{4}{3}\frac{Ε}{1-v^{2}}R^{1/2}$$

Equation (3) is also valid for spherical indenters but for small indentation depths (a typical limit is h<R/10 [24]). For spherical indenters and big indentation depths [24]

(4) $$a=\frac{4}{3}\frac{Ε}{1-v^{2}}R^{1/2}Z$$

In Equation (4),

(5) $$Z=c_{1}+\sum_{M=2}^{N}\frac{3}{2Μ}c_{M}R^{\left(\frac{3}{2}-M\right)}h^{M-3/2}$$

The values of coefficients $c_{1},c_{2},\ldots ,c_{N}$ can be found in [24]. Models for indenters with arbitrary shapes can also be found in [5]. 

As already mentioned, the equations presented in this section are valid for elastic half-spaces; however, they are extensively used when testing biological samples to create Young’s modulus maps [17,25]. A Young’s modulus map is created when multiple force–indentation curves are obtained on a selected region [26,27,28]. A typical illustration of the method is shown in Figure 2c, where the selected area has been divided into 8 × 8 = 64 nanoregions. A force–indentation curve is obtained in each nanoregion, and the experimental data is fitted to the appropriate model of contact mechanics depending on the indenter’s shape (Figure 2d). Assuming that the sample’s Poisson’s ratio is known (the Poisson’s ratio varies in the range 0≤v≤0.5, and usually is considered equal to 0.5 when testing biological materials in a liquid environment), the Young’s modulus of each nanoregion can be determined as a fitting parameter. A typical Young’s modulus map on a fibroblast using a conical indenter (with a half angle equal to 25° and cantilever’s spring constant 0.01 Nm^−1^) is presented in Figure 2e. The map was created using open-access nanoindentation data from the AtomicJ repository [29]. It must be noted that only the first 800 nm of the force-indentation data was taken into account to avoid the possibility of a substrate effect. The tested region is 50 μm × 50 μm, and the number of force -indentation curves used to create the map was 1024 (32 × 32). In addition, the histogram ‘probability–Young’s modulus’ is presented in Figure 2f. The term probability represents the number of occurrences of each Young’s modulus value on the tested area, normalized (i.e., as a fraction of 100).

Since biological samples are not infinite at the *x*-*y* plane and also are highly heterogeneous (as clearly shown in Figure 2e,f), significant questions arise regarding the rationality of this approach. In particular, the first question is related to the dimensions of the material with respect to the AFM tip. In most cases, the sample is significantly bigger compared with the nanometer-sized tip; thus under this perspective, the elastic half-space approximation is valid. For example, the map presented in Figure 2e was obtained using a sharp conical tip (tip radius ~20 nm) [29], and the maximum indentation depth was 800 nm. Thus, the dimensions of each tested region were several orders of magnitude smaller than the dimensions of the fibroblast [29]. It is also significant to note that modifications of classic Hertzian equations have been proposed for linear elastic samples with other geometries (for example, the case of collagen fibrils that can be modeled as cylindrical samples [30]). 

The second, and most important, question is related to the heterogeneity of biological materials. In particular, is it rational to apply equations that are valid for homogeneous materials on biological samples? To provide an answer, assume an indentation experiment using a parabolic indenter. The heavily deformed area is in the same order of magnitude as the contact diameter $2r_{c}$ (where $r_{c}=\sqrt{2Rh})$ [23,31]. Thus, the order of magnitude of the elastic deformation is $\varepsilon \approx h/\left(2r_{c}\right)$. In addition, the order of magnitude of the stress is $\sigma =Ε\varepsilon \approx Εh/\left(2r_{c}\right)$. Hence, the force applied to the sample is [31]:

(6) $$F=\sigma A\approx \frac{Εh}{2r_{c}}\pi r_{c}^{2}=\frac{\pi }{\sqrt{2}}Ε\sqrt{R}h^{3/2}$$

Equation (6) is comparable to the exact result [31],

(7) $$F=\frac{4}{3}\frac{Ε}{1-v^{2}}R^{1/2}h^{3/2}$$

Assuming v=0.5, they differ only by a factor of approximately 1.25 [31]. The same approximation can be also applied to other geometries. For a conical indenter, $r_{c}=h\tan\left(\theta \right)$ and the deformation is estimated, $\varepsilon \approx h/\left(2r_{c}\right)=1/\left(2\tan\left(\theta \right)\right)$. The order of magnitude of the stress, in this case, is σ=Εε≈Ε/(2tan(θ)). Thus, the force applied to the sample is [31]:

(8) $$F=\sigma A\approx \frac{E}{2\tan\left(\theta \right)}\pi r_{c}^{2}=\frac{\pi }{2}\tan\left(\theta \right)Εh^{2}$$

The exact result is:

(9) $$F=\frac{2}{\pi }\frac{Ε}{1-v^{2}}\tan\left(\theta \right)h^{2}$$

Equation (8) is comparable to Equation (9) (assuming v=0.5 they differ by a factor of approximately 1.85). The significance of Equations (6) and (8) is that they show that the calculated Young’s modulus is in the same order of magnitude as the exact result by considering that only a small portion of the material “is affected” (i.e., a heavily deformed area with contact $2r_{c}$). For example, as already mentioned, in Figure 2e, 32×32=1024 measurements are presented. The tested area is equal to $50\;\mu m\;\times 50\;\mu m=2500\;{\mu m}^{2}$. Thus, each Young’s modulus value refers to an area equal to $2.44\;{\mu m}^{2}$. The heavily deformed area for each measurement is $\pi r_{c}^{2}=\pi {\left(h_{c}\tan\left(\theta \right)\right)}^{2}=\pi {\left(\frac{2h_{\max}}{\pi }\tan\left(\theta \right)\right)}^{2}=0.177\;{\mu m}^{2}<<2.44\;{\mu m}^{2}$. Thus, it is a rational approximation to create Young’s modulus maps using classic equations provided by Hertzian mechanics (it is rational to consider small nanoregions and apply Equations (7) and (9) for the mechanical characterization of heterogeneous materials at the nanoscale). However, the mesh size when obtaining a Young’s modulus map is important; the contact area between the tip and the sample should be significantly smaller compared with the mesh size of the Young’s modulus map in order to approximate the tested region as an elastic half-space.

In addition, it is significant to note that the equations presented in this paper can be modified for indentation experiments on very thin samples. In the case that the sample is not bonded to the substrate [32],

(10) $$F=F_{\mathrm{Hertz}}\left[1+0.884\chi +0.781\chi^{2}+0.386\chi^{3}+0.0048\chi^{4}\right]$$

For bonded samples [32],

(11) $$F=F_{\mathrm{Hertz}}\left[1+1.133\chi +1.283\chi^{2}+0.769\chi^{3}+0.0975\chi^{4}\right]$$

In Equations (10) and (11) $F_{\mathrm{Hertz}}$ is provided by Equation (7) and $\chi =\sqrt{Rh}/D$ (D is the sample’s thickness).

Young’s modulus maps have been extensively used in the literature for the nanomechanical characterization of biological materials. For example, when using this approach, the changes in the mechanical properties of the dermis that occur during tissue remodeling or skin aging can be easily monitored [33]. Young’s modulus maps were created using the classic Hertzian approach (Equation (7)) and revealed that Young’s modulus varies between 0.1 KPa to 10 KPa depending on the body area and dermal layer [33]. Another application is the recording of the ultrastructural and nanomechanical properties of arterial tissues [34]. The tissue stiffness has been explored using the AFM indentation method. Young’s modulus maps of aorta tissue stiffness in its three constitutive layers (i.e., tunica intima, media, and adventitia) can reveal its mechanical properties. AFM with large and ultrasharp tips was used to explore tissue stiffness at two scales. Spherical probes for experiments at the micro-scale (tip radius ~2 μm) and sharp probes for experiments at the nanoscale (tip radius ~2 nm) were used [34]. Hertzian mechanics has also been used for data processing regarding experiments in individual collagen fibrils or collagen fibrils under the influence of external factors [3,4]. For example, the Young’s modulus of collagen fibrils decreases as a function of glycation, whereas the elastic modulus of collagen scaffolds increases [35].

Concluding, a final question arises; are there any limitations of typical Young’s modulus maps? In our opinion, the answer is positive. The major drawback of typical Young’s modulus maps (as the one presented in Figure 2e) is that they present the mechanical variability over the *x*-*y* plane. In other words, the depth-dependent variability (variability at the *z*-axis) is not considered. When typical Young’s modulus maps are presented, it is assumed that each nanoregion has a specific Young’s modulus that is independent of the maximum indentation depth (under the condition that there is no substrate effect). This is a significant limitation since when testing the same material (e.g., the same cell) but using different maximum applied forces in each case (resulting in different maximum indentation depths), the results are also different [36]. The mechanical properties of biological samples are strongly depth-dependent. Thus, two different researchers can conclude different results for the same material. 

It must also be noted that Hertzian mechanics assumes a purely elastic response (i.e., a sample that behaves as an elastic half-space). Even though biological samples at the nanoscale present a viscoelastic behavior, it has been proven that typical loading force indentation curves are well-fitted to equations arising by Hertzian mechanics (the effects of viscosity are negligible for the loading curve) [37]. It is important to note that Hertzian mechanics is the most extensively used theory for fitting force curves in the literature [38,39,40,41,42,43,44]. Alternatively, the theory developed by Ting [38] for the indentation of a linear viscoelastic half-space by a rigid axisymmetric indenter can be used. The viscoelastic behavior is dominant for high indentation rates; there is a significant hysteresis between the loading and the unloading curves [37]. There is a significant number of publications focusing on the viscoelastic properties of soft biological samples in the literature [45,46,47,48,49,50,51,52,53,54,55,56,57,58]. For example, Darling et al. derived a viscoelastic solution for small indentation depths on an incompressible, homogeneous, and isotropic material using a spherical indenter [46]. According to this model, the force applied on the viscoelastic materials is provided below [46]:

(12) $$F\left(t\right)=\frac{4R^{1/2}h^{3/2}E_{R}}{3\left(1-v\right)}\left(1+\frac{\tau_{\sigma }-\tau_{\varepsilon }}{\tau_{\varepsilon }}e^{-t/\tau_{\varepsilon }}\right)$$

In Equation (12), $\tau_{\sigma }$ and $\tau_{\varepsilon }$ are the relaxation times under constant load and deformation, respectively. The force–indentation data can then be fitted to Equation (12) to calculate three parameters, $k_{1}=E_{R}$, $k_{2}=E_{R}\frac{\left(\tau_{\sigma }-\tau_{\varepsilon }\right)}{\tau_{\varepsilon }}$ and $\mu ={Ε}_{R}\left(\tau_{\sigma }-\tau_{\varepsilon }\right)$, where $k_{1}$ and $k_{2}$ are the Kelvin spring elements, and μ is the apparent viscosity. In conclusion, the instantaneous and Young’s moduli are calculated as follows:

(13) $$E_{0}=E_{R}\left(1+\frac{\tau_{\sigma }-\tau_{\varepsilon }}{\tau_{\varepsilon }}\right)$$

(14) $$E=\frac{3}{2}E_{R}$$

Another approach was recently presented by Efremov et al. [37], based on the Ting’s model. Ting’s model provides a solution for the indentation of a linear visco-elastic half-space when using a rigid axisymmetric indenter for any load history. The model includes both loading and unloading parts of the force indentation curves. For rigid spherical indenters [37],

(15) $$F\left(t,\;h\left(t\right)\right)=\{\begin{array}{c} \frac{4\sqrt{R}}{3\left(1-v^{2}\right)}\int_{0}^{t}E\left(t-\xi \right)\frac{\partial \delta^{3/2}}{\partial \xi }d\xi ,\;0\leq t\leq t_{m}\\ \frac{4\sqrt{R}}{3\left(1-v^{2}\right)}\int_{0}^{t_{1}\left(t\right)}E\left(t-\xi \right)\frac{\partial \delta^{3/2}}{\partial \xi }d\xi ,\;t_{m}\leq t\leq t_{ind} \end{array},$$

(16) $$\int_{t_{1}\left(t\right)}^{t}E\left(t-\xi \right)\frac{\partial \delta }{\partial \xi }d\xi =0$$

In Equations (15) and (16), F is the applied force, t is the time (assuming that t=0 is the moment of the initial contact), $t_{m}$ is the duration of the loading phase, $t_{ind}$ is the duration of the complete indentation cycle, $t_{1}$ is the auxiliary function determined by the Equation (16), E(t) is the Young’s relaxation modulus and ξ is the dummy integration variable. A time-dependent E(t) is a decaying function that can be described by rheological models, like the standard linear solid (SLS) and power-law rheology (PLR) models. In particular,

(17) $$E\left(t\right)=E_{\infty }+\left(E_{0}-E_{\infty }\right)e^{-\frac{t}{\tau }},$$

(18) $$E\left(t\right)=E_{0}\left(1+\frac{t}{t^{\prime }}\right)e^{-a},$$

The SLS model is characterized by the instantaneous modulus ($E_{0}$), the long-term modulus ($E_{\infty }$) and the relaxation time (τ). The PLR model is characterized by the instantaneous modulus and the power law exponent a. If a=0 there is solid-like behavior; if a=1 there is a fluid-like behavior [37].

Using these approaches, the mechanical mapping of soft biological samples with respect to the indentation rate can be achieved (see, for example, [54]). However, when modeling the sample as a linear viscoelastic half-space, the same drawback regarding the derivation of the depth-dependent mechanical properties also applies. Thus, a mechanical mapping at the *x*-*y* plane and for different indentation rates is possible, but the mechanical variation towards the *z*-axis is not recorded. Thus, in Section 3, methods for obtaining the depth-dependent mechanical properties of biological samples are presented.

## 3. Depth Dependent Mechanical Properties

**Figure 3:**
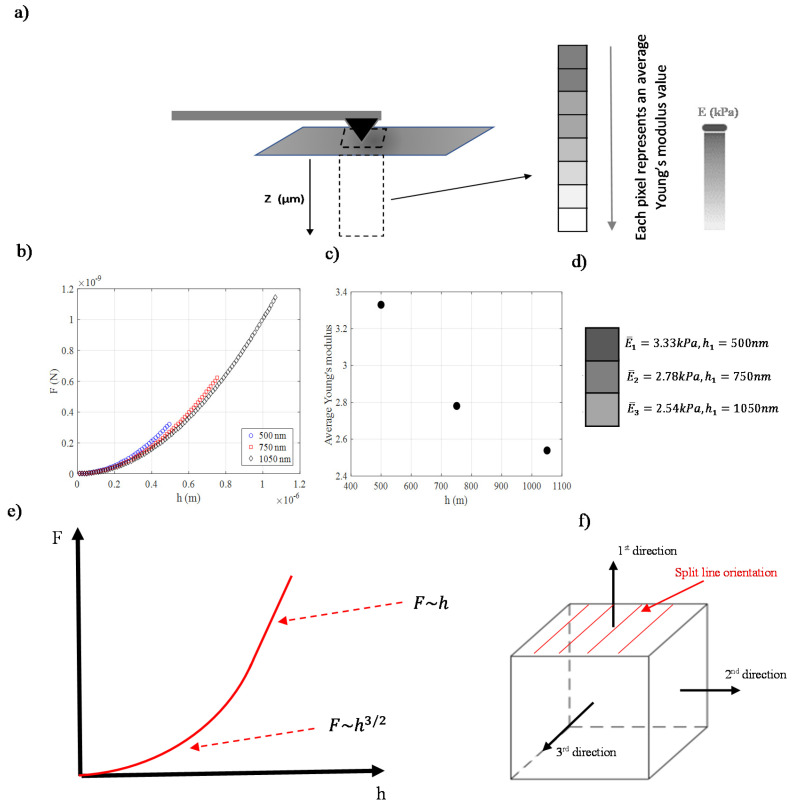
(**a**) Illustration of a nanoindentation experiment on a specific point. The depth-dependent mechanical properties at a specific nanoregion can be revealed by using this approach. If multiple points are tested, a depth-dependent Young’s modulus map can be created. (**b**) Three fitted curves for the same force indentation data (using Equation (9)) but for different domains (0≤h≤500 nm, 0≤h≤750 nm, 0≤h≤1050 nm). (**c**) The average Young’s modulus values for each domain. (**d**) Each value is represented by a colored pixel. (**e**) When using a spherical indenter on an elastic half-space the applied force is proportional to $h^{3/2}$ for small indentation depths and to h for big indentation depths. Thus, if the data are fitted to Equation (7), a ‘pseudo-softening’ behavior is recorded. (**f**) Determination of the depth-dependent anisotropy of the extracellular and pericellular matrices of articular cartilage.

As already mentioned, the mechanical properties of biological samples are depth dependent. There are several methods to obtain quantitative data regarding the depth-dependent behavior of soft samples at the nanoscale. The simplest approach is to calculate the ‘relative’ Young’s modulus value for each F, h data set. In particular, Equations (7) and (9) are usually used to calculate Young’s modulus for each pair of force–indentation values. Using this approach, a ‘relative’ Young’s modulus with respect to the indentation depth graph is constructed, also mentioned as the ‘pointwise modulus’. For example, Pogoda et al. used this approach on healthy human skin fibroblasts and on two human cancer cell lines, the WM35 (primary cutaneous melanoma cell line from the radial growth phase) and the A375 (a metastatic melanoma cell line) [59]. Their results showed a ‘power-law’ decrease of the relative Young’s modulus as the indentation depth increases. For big indentation depths, the relative Young’s modulus tends to a limit value [59]. Another method is to perform several fittings on the same force–indentation data for different domains. For example, for $0\leq h\leq h_{1}$, $0\leq h\leq h_{2}$, …, $0\leq h\leq h_{\max}$. The procedure is presented in Figure 3a. Three fitted curves from the same force indentation data obtained on a specific point on a fibroblast are shown (Figure 3b). The first fit was performed on the domain 0≤h≤500 nm, the second on the domain 0≤h≤750 nm and the third on 0≤h≤1050 nm. The Young’s modulus value for each case resulted in 3.33 kPa, 2.78 kPa and 2.54 kPa, respectively (Figure 3c,d). Using this approach, the ‘apparent Young’s modulus’ for different indentation depths can be constructed. This is an accurate approach for deriving the depth-dependent mechanical properties at the nanoscale. In particular, it has been recently shown that highly heterogeneous materials can be considered as the sum of a big number of homogeneous elementary slices [60]. Thus, when fitting the force indentation data to Equations (7) and (9), the calculated Young’s modulus is the average Young’s modulus ($\bar{Ε}$) of these slices [60]. In particular, when using a parabolic indenter,

(19) $$F=\frac{4}{3}\frac{\bar{Ε}}{1-v^{2}}R^{1/2}h^{3/2}\;$$

In addition, for a conical indenter,

(20) $$F=\frac{2}{\pi }\frac{\bar{Ε}}{1-v^{2}}\tan\left(\theta \right)h^{2}$$

Assuming that the sample can be considered as the sum of N narrow homogeneous slices, the average Young’s modulus is defined as follows:

(21) $$\bar{E}=\frac{1}{h}\sum_{i=1}^{N}E_{i}\Delta h$$

In Equation (21), $E_{i}$ is the Young’s modulus of a narrow slice at an arbitrary depth. Assuming that N→∞, Equation (21) is modified as follows:

(22) $$\bar{E}=\frac{1}{h}\overset{h}{\underset{0}{\int }}E\left(y\right)dy,$$

In Equation (22) E(y) indicates the depth-dependent Young’s modulus function. Using this approach, it is also easy to create depth-dependent Young’s modulus maps [60]. The calculation of the average Young’s modulus for different domains can also be performed by assuming an ‘equivalent nanoindentation experiment’ on an elastic half-space using the same maximum indentation depth and the same work done by the indenter as for the actual experiment [61]. Thus, the depth-dependent mechanical properties of a biological sample can be described by the average Young’s modulus function with respect to the indentation depth, $\bar{E}=f\left(h\right)$ [61]. Each nanoregion on a biological sample should have a unique $\bar{E}=f\left(h\right)$ function [61]. In particular, assume a hypothetical ‘energy equivalent’ problem (i.e., an indentation experiment using the same indentation depth and the same work done by the indenter) on a linear elastic sample. The force on the real sample should depend on the indenter’s properties, the varying Young’s modulus, and the indentation depth, $F_{\mathrm{real}}=cf\left(E,h\right)$, where c is a constant factor that depends on the indenter’s properties. Thus, the average force value for a specific maximum indentation depth and for a specific indenter depends on the average Young’s modulus, $F_{\mathrm{ave}}=g\left(c,\bar{E},h\right)$. 

In addition, for the equivalent real elastic material, $F_{\mathrm{ave}}=f\left(c,E,h\right)$. Since it has already been proven that the average force in the two aforementioned cases is the same, the Young’s modulus of the ideal linear material provides the actual average Young’s modulus of the sample [61]. In addition, for different values of indentation depth, the average Young’s modulus will also be different. Thus, the depth-dependent mechanical properties of a biological sample can be described by the average Young’s modulus function with respect to the indentation depth, $\bar{E}=f\left(h\right)$ [61]. To derive the $\bar{E}=f\left(h\right)$ function, the area under the force–indentation graph is separated into a wide number of parts. The work done by the indenter is calculated for each part that includes the previous ones. Subsequently, the graph of the average Young’s modulus with respect to the indentation depth is constructed, and the data are fitted to a representative function $\bar{E}=f\left(h\right)$ [61]. Using the aforementioned methodology, it has been recently presented that the average Young’s modulus for any cell type is provided by the following function $\bar{E}=ah^{b}+c$ where a,b,c are fitting factors (a,c>0 and b<0) [61]. The results following this approach are in good agreement with the results presented by Pogoda et al. [59]. 

It is also important to mention that an interesting mathematical model for describing the depth-dependent mechanical properties of cells was recently derived by Ding et al. [62]. In their model, a Finite Element Simulation analysis that considers the surface tension effects was used. The model was compared with experimental results obtained from normal and cancer breast cells [63], skin melanoma cells [64], cutaneous melanoma cells [59], fibroblasts treated with cytochalasin D (which is an inhibitor of actin polymerization and consequently significantly alters cell’s cytoskeleton) [59], and living erythrocytes [59]. The model’s prediction was in good agreement with the experimental data. In particular, the ‘power-law’ decrease of the calculated Young’s modulus as the indentation depth increases (which tends to a limit value for big indentation depths) was also observed in this case.

For indentations using a parabolic indenter, the applied force with respect to the indentation depth is presented below [62]:

(23) $$F=\frac{4}{3}\frac{E}{1-v^{2}}R^{1/2}h^{3/2}\left[1+a_{S}{\left(\frac{s}{\sqrt{Rh}}\right)}^{\beta_{S}}\right]$$

In Equation (23), $a_{s}=0.88\pm 0.0037$ and $\beta_{s}=0.87\pm 0.0034$ are two fitting parameters. In addition, $\frac{s}{\sqrt{Rh}}$ is a dimensionless length ($0.1\leq \frac{s}{\sqrt{Rh}}\leq 10$ [62]). Thus, the apparent Young’s modulus is derived as follows:

(24) $$E_{app.}=E\left[1+a_{S}{\left(\frac{s}{\sqrt{Rh}}\right)}^{\beta_{S}}\right]$$

For conical indentations [62],

(25) $$F=\frac{2}{\pi }\frac{E}{1-v^{2}}\tan\left(\theta \right)h^{2}\left[1+a_{c}{\left(\frac{s}{h\tan\left(\theta \right)}\right)}^{\beta_{c}}\right]$$

In Equation (25), $a_{c}=0.95\pm 0.0097$ and $\beta_{c}=0.92\pm 0.0088$ are two fitting parameters and, $\frac{s}{h\tan\left(\theta \right)}$ is a dimensionless length ($0.1\leq \frac{s}{h\tan\left(\theta \right)}\leq 10$ [62]). Thus, the apparent Young’s modulus, in this case, is derived as follows:

(26) $$E_{app.}=E\left[1+a_{c}{\left(\frac{s}{h\tan\left(\theta \right)}\right)}^{\beta_{c}}\right]$$

In addition, another new method for measuring the mechanical properties of soft heterogeneous materials at the nanoscale has been recently developed, namely the Trimechanic theory for general elastic response [65]. Materials with depth-dependent mechanical properties are assumed to exhibit a zone-wise pattern with a cone-like shape of the indenting tip. Within a specific zone, the mechanical properties are similar. The restoring force at the total indentation depth can be expressed as a sequence of force segments [65]:

(27) $$\begin{array}{c} F_{T}\left(h\right)=\sum_{i=1}^{m}\int_{Z_{i-1}}^{Z_{i}}{F^{\prime }}_{T}dZ=F_{T}\left(Z_{j-1}\right)+\sum_{i=j}^{m}\int_{Z_{i-1}}^{Z_{i}}{F^{\prime }}_{T}dZ=F_{T}\left(Z_{m-1}\right)\\ +\int_{Z_{m-1}}^{h}{F^{\prime }}_{T}dZ \end{array}$$

According to Equation (27), the limits of integration define a zone-wise region of the indented depth. In addition, for indentation depth equal to zero, $F_{T}=0$. For an arbitrary zone j, $F_{T}$ equals to a composite of three force components:

(28) $$F_{T}\left(Z\right)=F_{T}\left(Z_{j-1}\right)+{F^{\prime }}_{T}\left(Z_{j-1}\right)\times \left(Z-Z_{j-1}\right)+\int_{Z_{j-1}}^{Z}[{F^{\prime }}_{T}\left(y\right)-{F^{\prime }}_{T}\left(Z_{j-1}\right)]dy$$

In Equation (28), the first term is the force measured at the sub-surface of the zone $\left(Z_{j-1}\right)$, and is symbolized as $F_{C}$ (this is the force against the indenting tip when using the classic approaches). The second term is a Hookean force $\left(F_{H}\right)$, with a proportional constant of ${F^{\prime }}_{T}\left(Z_{j-1}\right)$. The third term satisfies the initial boundary conditions for applying Sneddon’s model. The three terms in Equation (28) introduce three nanomechanical modes, (trimechanic theory). Thus, the applied force can be decomposed into three factors, the depth impact $\left(F_{C}\right)$, the Hookean $\left(F_{H}\right),$ and the tip-shape $\left(F_{S}\right)$ factor. The trimechanic theory depicts how the nanomechanical properties vary with depth. 

Finally, another nanocharacterization approach towards the *z*-axis uses the work done by the indenter per unit volume [36]. It has been previously shown that for an elastic half-space, when using a conical indenter, the work per unit volume equals:

(29) $$\frac{W}{V}=\frac{\pi E}{4\left(1-v^{2}\right)\tan\left(\theta \right)}$$

Thus, for material with softening behavior the W/V ratio should decrease as the indentation depth increases. On the contrary, for a material with stiffening behavior, the ratio W/V increases as the indentation depth increases. The major advantage of this method is that it does not require a fitting procedure. Thus, the mechanical properties can be determined faster, enabling real-time analysis. On the contrary, a significant limitation is that it cannot be easily applied to other geometries (e.g., for parabolic indenters). The reason is that the ratio W/V is not constant for elastic half-spaces [36],

(30) $$\frac{W}{V}=\frac{32}{15}\frac{E}{1-v^{2}}R^{-1/2}h^{1/2}$$

However, it is also significant to note that a recorded change in the mechanical properties could be the cause of the misuse of contact mechanics equations. For example, Equation (7) is valid for parabolic indenters, but it can be also applied when using spherical indenters for h≪R as already mentioned. The accurate expression that relates the applied force to the indentation depth for a spherical indentation was derived by Sneddon [66] and is provided below:

(31) $$F=\frac{E}{2\left(1-v^{2}\right)}\left[\left(r_{c}^{2}+R^{2}\right)ln\left(\frac{R+r_{c}}{R-r_{c}}\right)-2r_{c}R\right]$$

The indentation depth is related to the contact radius with the following equation [66]:

(32) $$ln\left(\frac{R+r_{c}}{R-r_{c}}\right)=\frac{2h}{r_{c}}$$

Nevertheless, an alternating equation was recently derived by Kontomaris and Malamou which directly correlates the applied force to the indentation depth [67]:

(33) $$F=\frac{4ER^{1/2}}{3\left(1-v^{2}\right)}h^{3/2}Z$$

In Equation (33), where the correcting factor Z would be such that

(34) $$\begin{array}{c} Z=c_{1}+\frac{3}{4}c_{2}R^{-1/2}h^{1/2}+\frac{3}{6}c_{3}R^{-3/2}h^{3/2}+\frac{3}{8}c_{4}R^{-5/2}h^{5/2}+\ldots \\ +\frac{3}{2N}c_{N}R^{\left(\frac{3}{2}-N\right)}h^{N-3/2} \end{array}$$

or,

(35) $$Z=c_{1}+\sum_{M=2}^{N}\frac{3}{2Μ}c_{M}R^{\left(\frac{3}{2}-M\right)}h^{M-3/2}\;\;,\;Z\leq 1$$

Thus, when fitting the data, obtained on an elastic half-space, to Equation (7) for big indentation depths (using a spherical indenter) the fitting parameter that is determined is $E_{app.}=EZ$, where $E_{app.}$ is the apparent Young’s modulus and E the real Young’s modulus. 

Since Z≤1, the calculated Young’s modulus is smaller than the real Young’s modulus. In particular, for $\frac{h}{R}=1.325$, Z=0.868, and for $\frac{h}{R}=4.951$, Z=0.606. Therefore, as the indentation depth increases, the apparent Young’s modulus decreases. The result is a ‘pseudo-softening behavior’. The force–indentation curve when using a spherical indenter for big indentation depths is presented in Figure 3e. 

For small indentation depths $F\;\sim \;h^{3/2}$, the data will follow a better fit via Equation (7). On the contrary, for big indentation depths law *F*~*h* is applicable for data in order to alleviate the problem of an indentation using a flat-ended punch, since the contact radius remains constant [24,67]. The depth-dependent mechanical properties are a result of the non-homogeneity of biological samples. 

In addition, biological samples are also anisotropic (anisotropy is the property of being directionally dependent, i.e., the opposite of isotropy, which means homogeneity in all directions). Therefore, it is also interesting to note that AFM can also reveal the depth-dependent anisotropy of the micromechanical properties of biological samples. In particular, McLeod et al. investigated the depth-dependent anisotropy of the extracellular and pericellular matrices of articular cartilage [68]. Their goal was not only to characterize the depth but also to investigate the directional dependence of the micromechanical properties of cartilage. Indentation experiments were performed in the three directions of each sample (see Figure 3f), and Young’s modulus maps for each case were constructed. In Figure 3f, the split line direction is also presented (split lines are indicative of the preferred collagen fiber orientation parallel to the articular surface [69,70]). Furthermore, one of the basic goals of AFM nanocharacterization is the accurate characterization of the cell’s membrane mechanical properties. In Section 4, the results presented in the literature regarding shallow indentations on cells are discussed. 

## 4. Shallow Indentations on Cells

One of the most representative categories of biological materials that have been thoroughly investigated using AFM is biological cells. Although classic equations from Hertzian mechanics considers the material to be three-dimensional, the approach cannot result in a 3D mechanical characterization via typical Young’s modulus maps (as per Section 2). In effect, it is assumed that each nanoregion that is indented has a constant Young’s modulus (Figure 2). Cells are very sensitive to the indentation depth due to local cytoskeletal or polymer brush elements [71,72,73]. An accurate model to estimate the membrane’s elastic properties, as already mentioned, was developed by Ding et al. [62]. For example, for spherical indentations, the apparent modulus is given by Equation (24). For very small indentation depths, Equation (24) results in the apparent modulus of the cell’s surface. Assuming that $a_{s}=0.88$ and $\beta_{s}=0.87$ and $\frac{s}{\sqrt{Rh}}=10$, $E_{app.}=7.5235E$, where E is the elastic modulus that describes the overall properties of the cell. Thus, the slope of the F=f(h) curve decreases as the indentation depth increases [62]. 

Another approach to estimating the elastic properties of a cell (for very low indentation depth) is based on the ‘brush model’ [71]. The brush layer is a nonelastic part of a cell that is better described as an entropic/steric brush. At the domain 0.1≤h/L≤0.8 (where L is the brush length), the force indentation data when using a spherical indenter follow the equation below [71]:

(36) $$F\left(h\right)\approx 50k_{B}TR^{*}N^{3/2}\exp\left(-\frac{2\pi h}{L}\right)L$$

In addition, when using a conical indenter (also at the domain 0.1≤h/L≤0.8) [71],

(37) $$F\left(h\right)\approx \frac{25}{\pi }k_{B}TN^{3/2}\tan^{2}\theta \exp\left(-\frac{2\pi h}{L}\right)L^{2}$$

In Equations (36) and (37), $k_{B}$ is the Boltzmann constant, T is the temperature, and θ is the cone’s semi angle. Furthermore,

(38) $$R^{*}=\frac{R_{\mathrm{indenter}}\cdot R_{\mathrm{cell}}}{\left(R_{\mathrm{indenter}}+R_{\mathrm{cell}}\right)R_{\mathrm{indenter}}}$$

The brush model is also important since it has been shown that cells can be described with a single elastic modulus if the pericellular brush layer is considered as a separate cellular structure. 

Another approach to acquiring information regarding the membrane elasticity of a cell is by using 2D contact mechanics models [74]. In case of cell membranes, the elastic energy can be described by Helfrich Hamiltonian [75]

(39) $$U=\frac{1}{2}k_{\mathrm{bend}}\int^{\;}{\left(c_{1}+c_{2}\right)}^{2}dA+\frac{1}{2}k_{\mathrm{stretch}}\frac{{\left(A-A_{0}\right)}^{2}}{A_{0}}$$

The first term of Equation (39) describes the bending energy ($k_{\mathrm{bend}}$ is the bending modulus), $c_{1},c_{2}$ are the membrane principal curvatures, dA is an elementary surface $k_{\mathrm{stretch}}$ is the membrane stretching modulus, and A and $A_{0}$ are, the perturbed and unperturbed surface areas of the membrane, respectively. This integral depends linearly on the indentation depth due to the inverse proportion between the principal curvatures and the local surface area [76]. 

Consequently, the relation of the force with the indentation depth is mostly based on the second term. This term represents the lowest order of the in-plane tension energy. As a result, the leading term of the indentation force is given below [74]:

(40) $$F=k_{\mathrm{stretch}}\frac{A-A_{0}}{A_{0}}$$

Using Equation (40) a description of the membrane protrusion due to tip indentation can be obtained. The model described above does not consider the contribution of attractive forces between the cell and the tip. In the case that the membrane of the cell does not adhere to the tip, the effect of tip geometry is small (before the tip reaches the inner cell components). Assuming the simplest case of a perfect conical indenter [74],

(41) $$F=k_{\mathrm{stretch}}\sqrt{\frac{h^{2}}{x^{2}}+1}$$

In Equation (41), x is the cone radius (it can be approximated by half cortex mesh size). Equation (41) can be fitted to a power law equation (Equation (1)), where 1≤m≤2. This is a possible explanation why for small indentation depths, the data is well fitted to classic equations from Hertzian mechanics, Equations (7) and (9). 

In conclusion, a determination of the mechanical properties of a cell’s membrane and, subsequently, the determination of a cell’s overall properties can provide information regarding the depth-dependent mechanical properties of cells leading to a complete spatial nanocharacterization. In Section 2, Section 3 and Section 4, the appropriate methodologies for retrieving the elastic properties of soft biological samples were presented. Section 5 focuses on hard biological samples, such as collagen fibrils.

## 5. Spatial Characterization of Collagen Fibrils

The elastic properties of single collagen fibrils at the nanoscale have been extensively studied in the past using the AFM nanoindentation method [77,78,79,80,81,82,83,84,85]. The equations that are being used for data processing (fitting the force indentation data) are presented in [30]. Many models have been proposed, such as the elastic interaction between a spherical indenter and an elastic half-space (when the diameter of the fibrils is significantly bigger compared with the AFM tip radius), the elastic interaction between two spherical solids, and the elastic interaction between a rigid sphere and a cylinder [30]. These models are simple extensions of Equation (7) with appropriate correction factors [30]. In addition, Vlassak and Barber’s model can also be used for anisotropic materials such as fibers at the nanoscale [86,87]. Another model for data processing when using sharp pyramidal indenters is the Oliver and Pharr method which accounts for an elastic-plastic interaction [88]. The Oliver and Pharr method is the most appropriate tool for hard biological samples (e.g., collagen fibrils) [8,83,88]. In this case, the unloading force–indentation data can be fitted to the following power law equation:

(42) $$F=a{\left(h-h_{f}\right)}^{m}$$

In Equation (42), $h_{f}$ is the final indentation depth of the contact impression after unloading and a, m are constants that depend on the indenter’s geometry. Usually, the exponential factor m varies between 1.2 and 1.6 for a Berkovich indenter [88]. Subsequently, the slope of the fitted curve is calculated at the maximum indentation depth (i.e., the stiffness $S={\frac{dF}{dh}|}_{h_{\max}}$) and the Young’s modulus can be calculated using the following equations:

(43) $$S=\beta \frac{2}{\sqrt{\pi }}E_{eff.}\sqrt{A}$$

(44) $$\frac{1}{E_{eff.}}=\frac{1-v^{2}}{E}+\frac{1-v^{2}}{E_{i}}$$

In Equation (43), β is a correction factor accounting for tip geometry (e.g., for a Berkovich tip usually ranges from 1.0226 to 1.085) and A is the contact area as a function of contact depth $h_{c}$. For a perfect Berkovich tip $A=24.5h_{c}^{2}$, and the contact depth is calculated as follows:

(45) $$h_{c}=h_{\max}-\varepsilon \frac{F_{\max}}{S}$$

The parameter ε can be calculated using the following equation:

(46) $$\varepsilon =m\left\{1-\frac{2\Gamma \left[\frac{m}{2\left(m-1\right)}\right]}{\sqrt{\pi }\Gamma \left[\frac{1}{2\left(m-1\right)}\right]}\left(m-1\right)\right\}\;$$

However, m=0.75 is a generally accepted value [88,89]. In addition, it is significant to also note that the Oliver & Pharr method is not the only approach in the literature for determining the Young’s modulus of hard samples such as collagen fibrils. The classic Hertzian approach has also been extensively used [37,90,91].

Using the indentation method, the radial Young’s modulus of the fibril on different nanoregions (overlapping and gap regions can be determined). Another recently presented approach is the determination of the tensile modulus of collagen fibril [92]. In particular, in this method, fibrils were deposited on a pre-stretched foil. Subsequently, the foil was released with the fibrils attached. The tensile modulus can be determined by AFM imaging by determining the buckling wavelength and the radius for each fibril. In particular, the wavelength (k=2π/λ) of the buckling pattern by two very similar equations is provided below [92]:

For in-plane buckling [93],

(47) $${\left(\frac{E_{\mathrm{buckling}}I}{{\bar{E}}_{s}}\right)}^{1/4}k={\left\{\frac{2\pi \left[\frac{1}{1-v_{s}}-\gamma -\ln\left(kR\right)\right]}{{\left[\frac{3-v_{s}}{1-v_{s}}-2\gamma -2\ln\left(kR\right)\right]}^{2}}\right\}}^{1/4}$$

For out-of-plane buckling [93],

(48) $${\left(\frac{E_{\mathrm{buckling}}I}{{\bar{E}}_{s}}\right)}^{1/4}k={\left\{\frac{2\pi \left[1-\gamma -\ln\left(kR\right)\right]}{{\left[3-2\gamma -2\ln\left(kR\right)\right]}^{2}}\right\}}^{1/4}$$

Equations (47) and (48) can be accurately used for a beam with cylindrical shape with radius R. $E_{\mathrm{buckling}}$ is the beam’s tensile modulus and ${\bar{E}}_{s}=\frac{E}{1-v_{s}^{2}}$ is the reduced modulus of the substrate ($E,\;v_{s}$ is the Young’s modulus and the Poisson ratio of the substrate respectively, $I=\left(\frac{\pi }{4}\right)R^{4}$ and γ=0.577 is the Euler’s constant) [94]. In summary, buckling under load can be used to determine the tensile modulus of nanometer-scale collagen fibrils from simple AFM topography images. By combining this approach with the classic AFM nanoindentation method, a complete mechanical characterization at the nanoscale can be achieved. 

**Figure 4:**
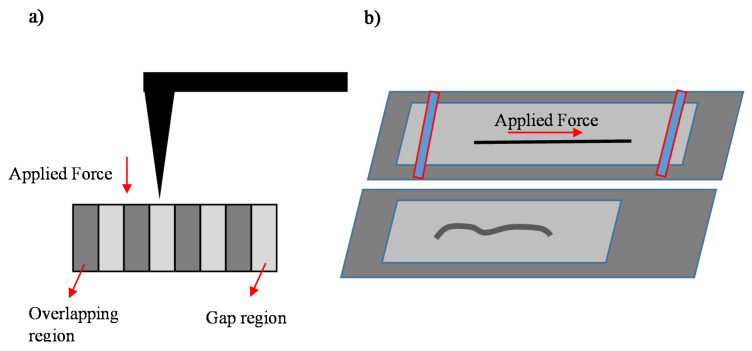
(**a**) Determination of the radial modulus using the AFM indentation method, (**b**) Determination of the tensile modulus using AFM imaging.

AFM nanoindentation method on a collagen fibril and the method for determining the tensile modulus are represented in Figure 4. In addition, methods for a three-dimensional characterization at the microscale will also be presented in Section 6.

## 6. 3D and 4D Nanomechanical Methods

**Figure 5:**
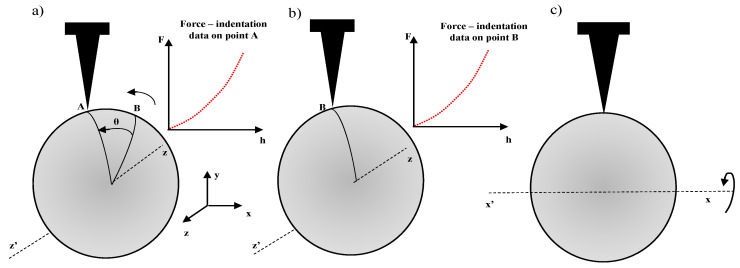
3D mechanical characterization at the microscale. (**a**,**b**) The sample is rotated along the *z*-axis and force indentation curves are obtained on points A and B. (**c**) The sample is rotated along the *x*-axis.

An important method for a complete spatial nanocharacterization of soft biological samples at the microscale was recently developed using acoustic manipulation and force microscopy [95]. In particular, an acoustically-driven manipulation device with a micro-force sensor was developed to freely rotate biological samples and quantify mechanical properties at multiple regions. Acoustic streaming, generated by exciting trapped microbubbles, allows the trapping of the samples and their re-orientation with respect to the force sensor [95]. In this method, the probe is pressed into the sample, and the resulting forces and indentation depths are recorded. The procedure is described in Figure 5 (it is assumed that the sample is spherical for simplicity). In Figure 5a,b, the sample is rotated along the *z*-axis, and multiple force indentation curves are recorded. For example, in Figure 5a, the force-indentation curve is obtained at point A, while in Figure 5b, the curve is obtained at point B. In Figure 5c, the sample is rotated along the *x*-axis, and the same procedure is repeated. Using this approach, a complete mechanical micro-characterization at the 3D level is performed.

It is important to note that with this approach, it is possible to determine the 3D inhomogeneity and anisotropy of the sample at the microscale. Another approach to mechanical characterization is the 4D approach, where the AFM tip performs continuous imaging and indentations on the sample using a specific applied force to monitor changes in the sample with respect to time. The continuous testing of the same area allows dynamic changes (i.e., changes with respect to time, as already mentioned) in the morphology of the area and the mechanical properties [96,97,98,99]. For example, in [97], the Young’s modulus distributions of liver endothelial cells under different protocols and with respect to time are presented.

## 7. Overcoming Challenges in AFM Nanocharacterization

As presented in this review paper, many different approaches have been presented up to date to provide a detailed characterization of the elastic properties of biological samples at the nanoscale. A typical summary of these methods is presented in Table 1. Typical Young’s modulus map is an accurate tool, as presented in Section 2; however, it can only provide the variation of the elastic properties at the *x*-*y* plane. In addition, when using different indentation depths, the resulting map is also different; therefore, it is difficult to conclude reproducible results. To overcome these limitations, new models that take into account the depth-dependent variability of biological samples have been derived (Section 3 and Section 4). In addition, groundbreaking techniques for a 3D micro-characterization or combined methods that use continuous imaging and mechanical mapping have been previously published. However, despite the ongoing innovations in the field, a complete 3D characterization at the nanoscale has not been achieved yet. Nevertheless, the depth-dependent techniques presented in Section 3 and Section 4 can be used in the future for the construction of 3D elastic maps at the nanoscale that will replace the classic 2D Young’s modulus maps. 

A complete 3D mapping at the nanoscale will probably provide significant information with respect to the characterization of complex biological samples and lead to easily reproducible results. The key to achieving a complete 3D nanocharacterization underlies two very recently developed theories; the extension of classical Hertzian mechanics for highly heterogeneous materials using the average Young’s modulus [60,61] and the Trimechanic theory for general elastic response [65]. These two theories can provide extended details regarding the depth-dependent elastic properties of biological samples, and if combined with the procedure for obtaining typical Young’s modulus maps at the nanoscale, a 3D nanocharacterization can be achieved. In addition, AFM is used for small scales and is appropriate to determine the depth-dependent mechanical properties near the surface. The most appropriate technique to use AFM and determine the mechanical properties through the thickness of the tissue is to collect serial sections and indent multiple sections for the *z*-dependent properties.

A 3D nanomechanical characterization may lead to groundbreaking results regarding the diagnosis of various diseases, such as cancer. As already mentioned in the introduction, a cancer diagnosis can be based on the different Young’s modulus distributions between normal/benign and cancer tissues. However, it is still unclear in the latest publications which should be the optimal indentation depth when testing a tissue sample. Thus, by creating 3D elastic properties maps, multiple Young’s modulus distributions for different indentation depths can be acquired. Thus, the ‘two peak’ stiffness distribution on cancer tissues can be monitored as the indentation depth increases. 

In addition, monitoring the elastic properties of malignant tissue and the properties of the surrounding tissues is crucial since slight local mechanical alterations could provide information regarding the metastasis procedure. A basic goal is to discover the paths used by cancer cells through metastasis based on the mechanical properties of the surrounding tissues. Thus, it will be possible to understand whether a metastasis procedure is in progress. Another significant application is the cancer prognosis. In particular, monitoring the 3D elastic properties of a malignant tissue under specific treatments is very important to evaluate the effects of those treatments. A 3D elastic properties map at the nanoscale could become a powerful tool at the hands of doctors, facilitating decision-making regarding the best personalized treatment procedure (for example, it will help surgeons to understand the exact amount of tissue that should be surgically removed). 

## 8. Conclusions

The nanomechanical characterization of biological samples at the nanoscale has provided valuable information with respect to the diagnosis of several diseases. Typical Young’s modulus maps are a strong tool. However, it is also important to provide a depth-dependent mechanical characterization when testing biological materials. New theoretical tools have been developed in this direction that will enable in the future complete spatial mechanical characterization at the nanoscale and enable AFM equipment to be used as a powerful clinical tool.

## Figures and Tables

**Table 1:** Methods for determining spatial variability of bio-samples elastic properties.

Method	Description
Young’s modulus maps	Determining the spatial variability of soft biological samples at the xy plane Theoretical framework: Hertzian mechanics
Depth-dependent mechanical properties	Relative elastic modulus Theoretical framework: Calculating E using each pair of F, h. Average Young’s modulus Theoretical framework: Considering the sample as a sum of N homogeneous slices. The average YM for a specific indentation depth is the average of the YM of these slices. Trimechanic theory Theoretical framework: The applied force can be decomposed to three factors, the depth impact (FC), the Hookean (FH) and the tip-shape (FS) factor. Work per unit volume Theoretical framework: The work done by the indenter per unit volume is calculated. When using a conical indenter, the depth-dependent mechanical behavior is revealed. Surface tension A model that considers the surface tension effects when determining the mechanical properties of cells. Theoretical framework: Extension of Hertzian theory (Equations (23)–(26))
Shallow Indentations	Brush model Theoretical framework: Model that considers the nonelastic part of the cell at very low indentation depths (Equations (36)–(38)). Determining the membrane elasticity of a cell Theoretical framework: Helfrich Hamiltonian (Equations (39)–(41)) Surface tension A model that considers the surface tension effects when determining the mechanical properties of cells. Theoretical framework: Extension of Hertzian theory (Equations (23)–(26))
Cylinder shaped samples	Spatial nanocharacterization. Theoretical framework: Hertzian fittings on force curves for calculating the radial elastic modulus combined with tensile modulus determination (Equations (47) and (48))
3D and 4D nanocharacterization	3D nanocharacterization. Theoretical framework: an acoustically driven manipulation device with a micro-force sensor was developed to freely rotate biological samples and quantify mechanical properties at multiple regions. 4D nanocharacterization. Theoretical framework: Construction of both stiffness and topography images. The continuous imaging of the same area allows the dynamic changes (i.e., changes with respect to time)

## Data Availability

Data from AtomicJ software are referenced in the manuscript, and a link is provided.
